# Video gaming facilitates adaptation to surgical exoscopes – a laboratory experiment

**DOI:** 10.1007/s00701-025-06589-2

**Published:** 2025-06-26

**Authors:** Adam Yousfi, Anni Pohjola, Ville Vasankari, Ahmad Hafez, Martin Lehecka

**Affiliations:** Department of Neurosurgery, Helsinki University Hospital, University of Helsinki, P.O. Box 266, FI-00029 Helsinki, Finland

**Keywords:** Exoscope, Neurosurgery, Surgical training, Video gaming

## Abstract

**Introduction:**

Digital 3D exoscopes are novel alternatives for operating microscopes in microneurosurgery. We studied the potential benefits of a background in video gaming on adaptation to exoscopic microsurgery. Such effects have not been reported before.

**Methods:**

Twenty preclinical medical and dental students with no surgical experience participated in our study. Eleven (gamer group) were proficient (> 1000 h) in video gaming, whereas nine (control group) had no to very little gaming experience (< 500 h). We developed a microsurgical training model for evaluating adaptation to exoscope use in a manual task. Each student completed the task thrice in a row. We evaluated handling of the exoscope, fine motor skills, and the duration of each performance.

**Results:**

Prior video gaming experience correlated with superior adaptation to the exoscope. Gamers moved and tilted the exoscope camera less often than the control group but nevertheless obtained good visuals. The gamer group also completed all tasks faster (median 10 min 14 s) than the control group (median 13 min 01 s). All students improved in their task completion times over the test period. Similar fine motor skills were observed in both the gamer and the control group.

**Conclusion:**

Prior experience in video gaming was associated with better adaptability to the exoscope. This may be due to superior 3D perception, acquired playing video games.

**Supplementary Information:**

The online version contains supplementary material available at 10.1007/s00701-025-06589-2.

## Introduction

Three-dimensional (3D) digital exoscopes are novel tools for the magnification and visualization of structures in neurosurgery [20, 28]. A robotic arm, equipped with a 3D digital camera, is controlled by a surgeon e.g., with a footpedal. The camera’s view of the surgical field is projected on a 3D monitor. Exoscope use in complex neurosurgery has led to outcomes comparable to the ones attained with a microscope [34, 40]. Exoscopes allow surgeons to maintain an ergonomic posture during surgery, while enabling accurate target visualization and a wide range of motion [27, 29, 42]. In addition, augmented reality may be integrated with exoscopes in the future [18].

Regardless of whether a surgeon utilizes an exoscope or a microscope, microsurgical operating technique is virtually identical. However, there are significant differences between the two magnifying devices themselves, and this requires relearning and adaptation in the case of experienced surgeons. As for novices, they may adapt similarly to some aspects of exoscopic microsurgery, but differently to other aspects as they do not have the burden of previous experience with surgical microscopes. Adaptability to the exoscope may also be influenced by other factors such as video gaming experience, however, systematic studies have not been conducted on this topic [2, 30].

We designed an experimental laboratory study to evaluate the effects of long-term video gaming on the adaptation to a surgical exoscope among beginners in microsurgery. Our hypotheses were: (1) prior video gaming experience will lead to superior adaptation to the exoscope, (2) improvement in exoscope utilization will be observed in all participants during one-hour training and (3) manual dexterity will be similar in both groups, but gamers will perform better overall due to efficient exoscope control.

## Materials and methods

### Participants

We recruited 20 preclinical first- and second-year medical and dental students (10 women, 10 men). None of them had prior experience in surgery/mirosurgery or exoscope use. All participants reported normal 3 d depth perception. Data collection was conducted between October 2023 to May 2024. All participants gave their informed consent to participation. We adhere to the Declaration of Helsinki.

### Study design

All participants performed the same microsurgical task thrice in a row. The performances were video recorded by the exoscope and saved on an external hard drive for further analysis.

The participants were divided into two groups. The control group (*n* = 9) consisted of students who had well under 500 h of experience in video gaming. The gamer group (*n* = 11) consisted of students who had played video games for over a thousand hours. The student with the least gaming experience from the gamer group had spent significantly more time gaming than the most experienced student from the control group. All students from both groups lacked prior experience in macro and microsurgery and had never used an exoscope nor an operating microscope.

### Test task

**Fig. 1:**
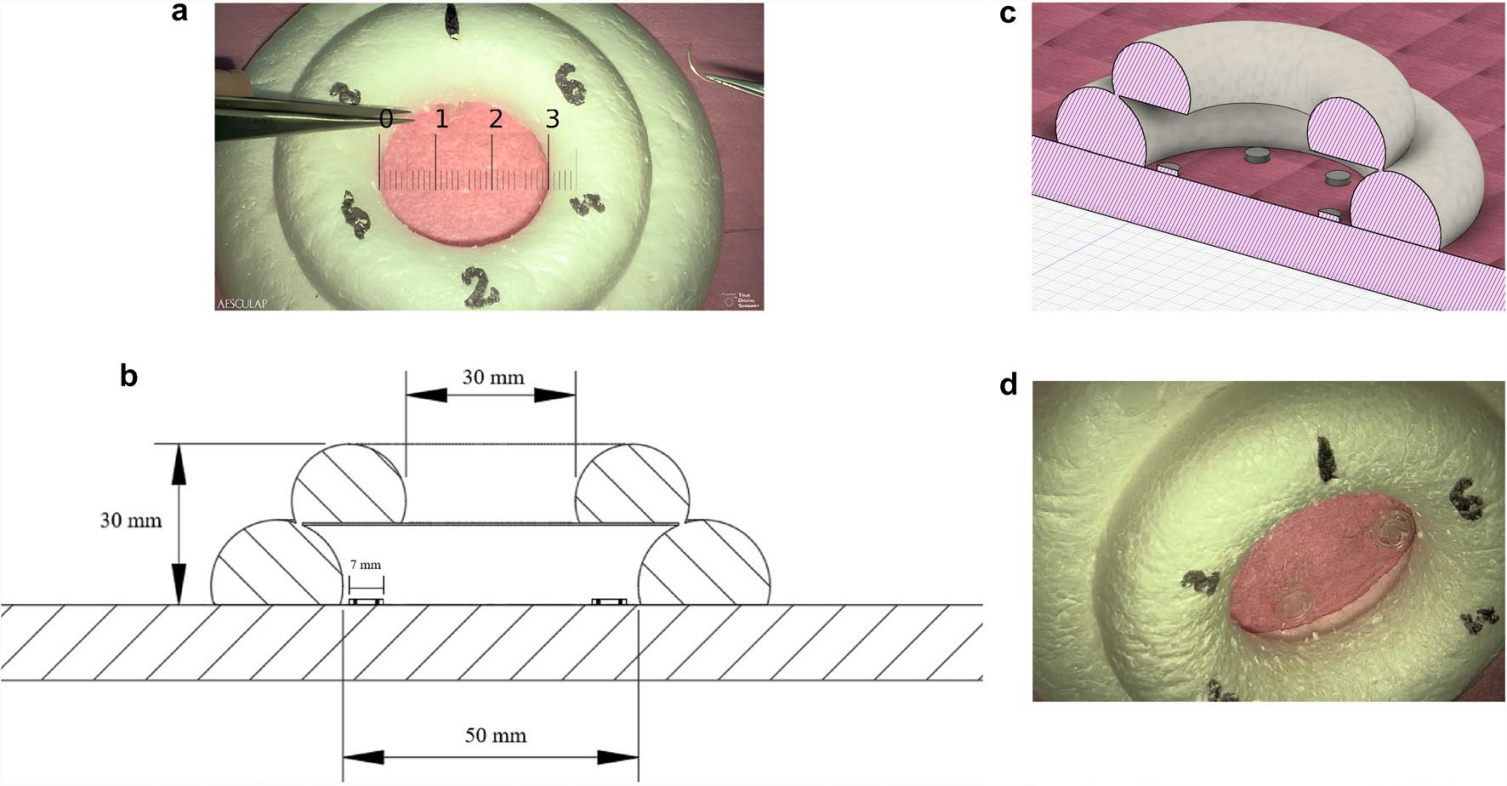
**a** The model from above [cm]. This image has been captured using the exoscope. **b** A 2D cross section of the model. Created using Autodesk Fusion. **c** A 3D cross section of the model. Created using Autodesk Fusion. **d** An oblique view of the model, showcasing the normally concealed target discs. This image has been captured using the exoscope

The model used in the test task was specifically developed to evaluate active exoscope use (camera motion and image adjustments) and fine micromotor skills. The model is represented in Fig. 1. It consists of two Styrofoam rings forming an inverted funnel with the orifice smaller than the base. The inner diameter of the upper Styrofoam ring torus measures 3 cm, the depth is 3 cm, and the diameter of the base where the discs are distributed is 5 cm. Inside, there are six symmetrically positioned plastic discs, 0.7 cm in diameter, attached to the base of the model. Each disc contains four, symmetrically positioned slits, through which a needle may pass. A disc is located beneath each number (1–6) visible in Fig. 1a.

The test task was performed with two identical jeweller’s forceps and a needle attached to 6–0 monofilament thread. The task was to pass the thread through all six discs in numerical order under the exoscope (Aesculap AEOS, BBraun). The participants were permitted to adjust the image (zoom and focus) and move the exoscope camera during the task as they wished. Each student was instructed to complete the task thrice in a row during the same training session. The participants were allowed to take a short break (1–2 min) in between the three rounds. The test tasks were preceded by a brief, on-location tutorial on exoscope use, and a few minutes of free-training. The participants had also been supplied beforehand with a video recording, which provided an overview of exoscope use and a model run of the test task (Video 1).

### Video analysis

All the video recordings were analysed by one researcher (AY) in a blinded order. Discrepancies were solved in accordance with two other researchers (AP, VVa). Video analysis was performed manually (VLC software).

**Fig. 2:**
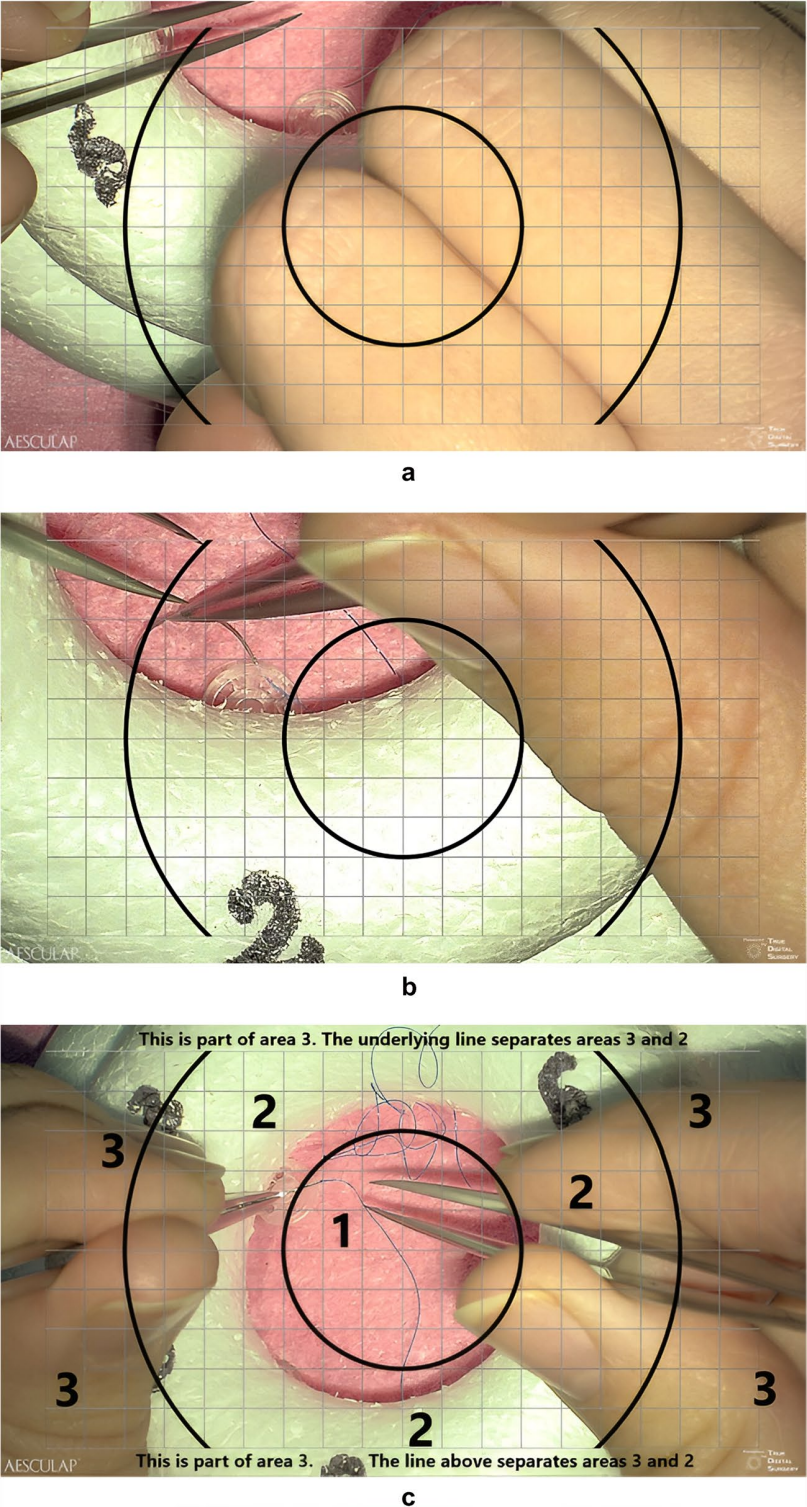
**a** Needle makes first contact with disc no. 2. This image has been captured using the exoscope. **b** Needle exits disc no. 2. This image has been captured using the exoscope. **c** On-screen areas. This image has been captured using the exoscope

Video analysis focused on two main areas: (a) handling of the exoscope and (b) fine motor skills. The actual parameters used for analysis are presented in Table 1, and Fig. 2 aids in understanding the parameters. Time was measured from the start of the test task (first movement of the exoscope) until the end of the task (needle passes through the last disc). Additional time stamps were taken for movement from each disc to the next one and passing through each disc.

**Table 1:** All variables

Total Time	Total duration of the task
Transition Time	Time taken to transition to a disc
Disc Entry Time	Time taken to pass through a disc
Camera Movement, Transition	Number of exoscope camera movements (movements in plane and tilting movements) when transitioning to a disc
Camera Movement, Disc Entry	Number of exoscope camera movements when passing through a disc
Disc Visibility	1/3: < 33% of the disc visible, 2/3: 33–66% visible, and 1 > 66% visible. Variable analysed twice for each disc, once when the needle first contacts the disc, and once when it exits the target disc (Fig. 2a and b)
Zoom	Size of unobstructed disc on screen (estimated by the number of squares in the overlaid grid (Fig. 2c)). Variable analysed twice for each disc, once when the needle first contacts the disc, and once when it exits the target disc (Fig. 2a and b)
Target Centrality	How well is the camera centred on the target? Which area of the overlaid grid, 1–3, contains the disc? (Fig. 2c). Variable analysed twice for each disc, once when the needle first contacts the disc, and once when it exits the target disc (Fig. 2a and b)
Needle Dropped	Number of times the needle was dropped
Instruments out of Field	Number of times that both instruments were out of the surgical area (areas 1 and 2 in Fig. 2c)

Due to some issues with equipment, we failed to record the test runs of one student from the control group and another from the gamer group. However, their performance times were available to us for analysis.

### Statistical analysis

Microsoft Excel was employed to produce tables and graphs. SAS statistical software version 8.3 was used for mixed model statistical analysis via the PROC MIXED procedure. Quantitative variables were handled as continuous. A p-value less than 0.05 was considered to indicate statistical significance.

## Results

All participants completed the last test task (median 10 min 58 s) faster than the first one (median 14 min 53 s). The gamer group completed all tasks faster (median 10 min 14 s) than the control group (median 13 min 01 s). Gamers moved and tilted the exoscope camera less often than the participants in the control group, *p* = 0.01, but nevertheless obtained similar visuals on the target discs. Gamers drifted out of the optimal viewing field with their instruments less often than the control group, *p* = 0.011.

### Timing

**Fig. 3:**
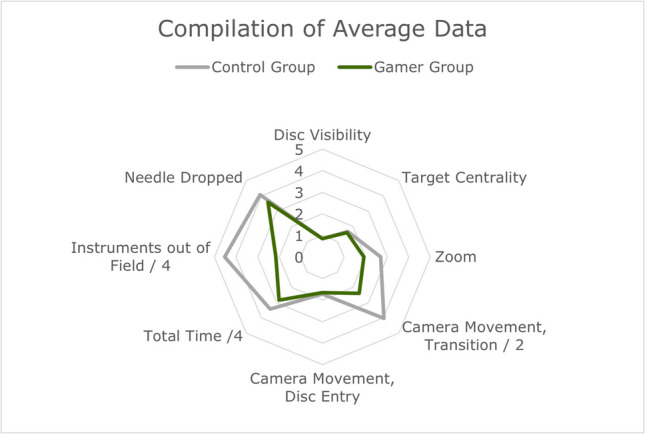
Synthesis of variable averages per all trials. See Table 1 for details on the variables. For the purposes of this figure, Camera Movement, Transition—values have been divided by two and Total Time and Instruments out of Field values with 4, to fit the data into the graph

The gamer group was faster than the control group both in completing the tasks and transitioning from disc to disc. For passing the needle through a disc there was no statistical difference between the groups. Both groups improved their times throughout the three test runs (Fig. 3). This improvement did not differ between the groups.

**Fig. 4:**
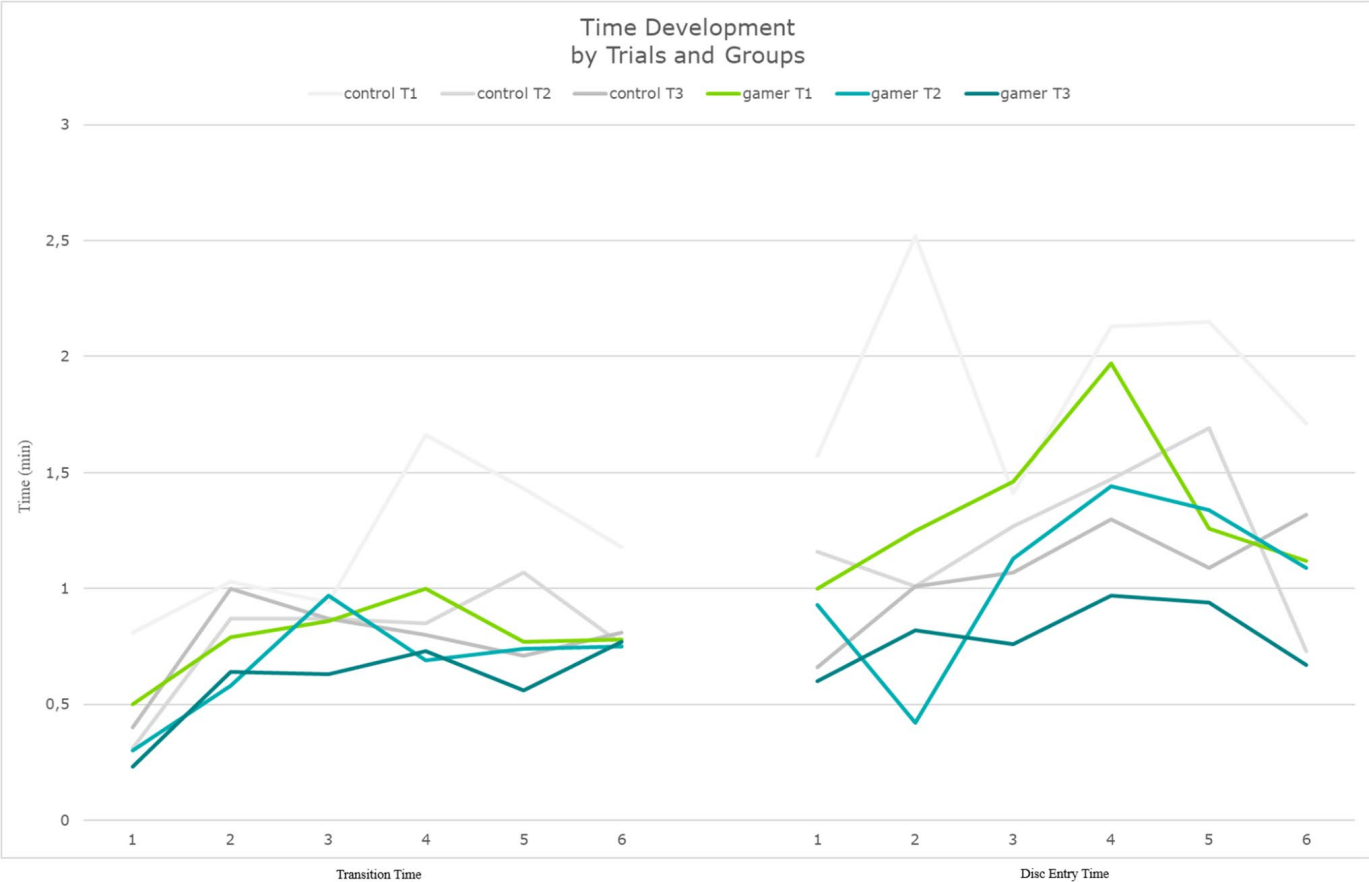
Transition time and disc entry time across trials, discs, and groups. Discs across all trials on the x-axis, time–values on the y-axis as minutes

The gamer group transitioned between discs faster than the control group (Fig. 4). Statistical significance was indicated both between groups, *p* = 0.04, and trials, *p* < 0.0001. Development did not differ significantly between the groups, *p* = 0.13.

The participants of both groups took similar time to pass the needle through discs. Statistical significance was not indicated between groups, *p* = 0.12, but was indicated between trials, *p* = 0.0003. Development did not differ significantly between groups, *p* = 0.44.

### Handling of the exoscope

**Fig. 5:**
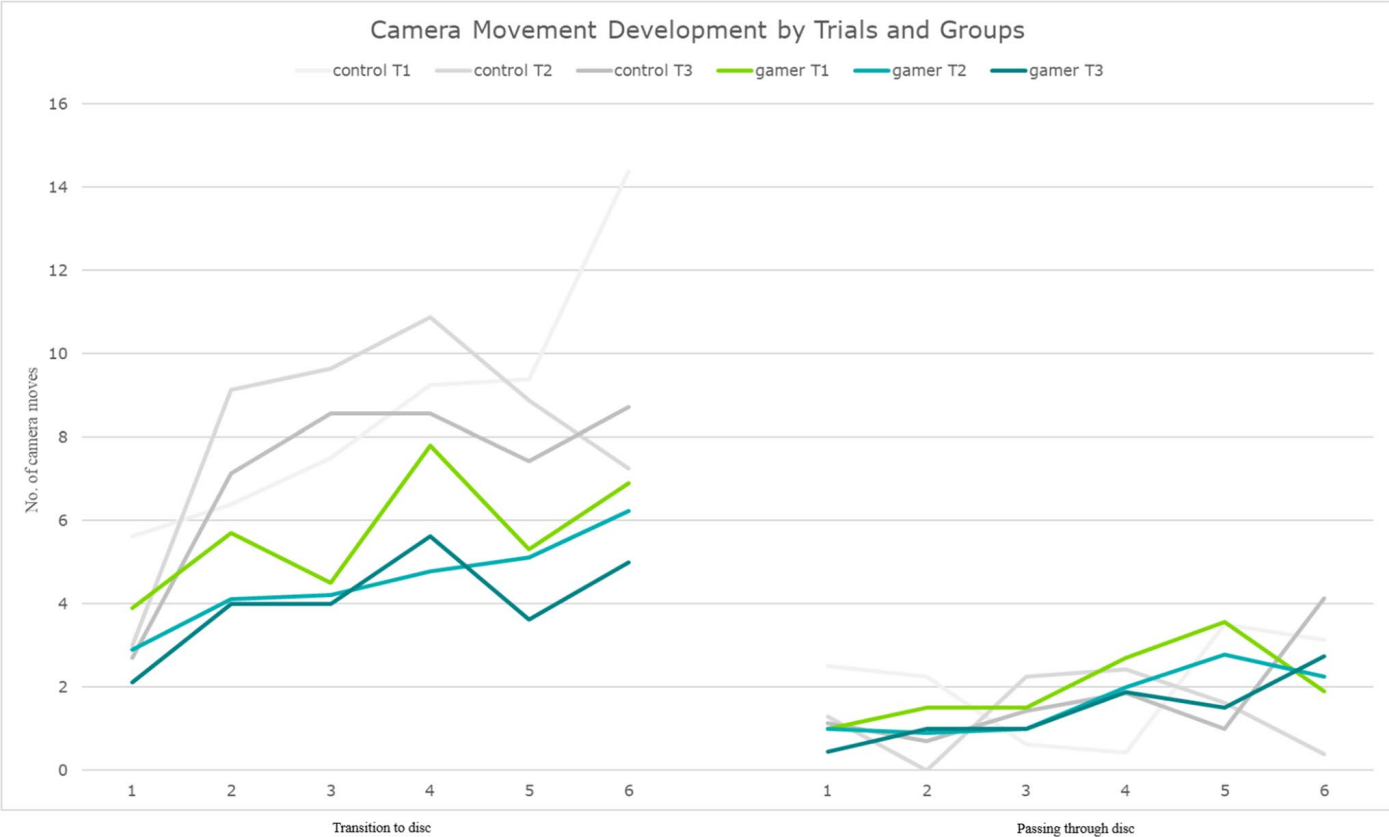
Camera movement development across trials, discs, and groups. See Fig. 1a for disc orientation. Discs across all trials on the x-axis, values on the y-axis. The higher the value, the more often the exoscope camera has been moved on average

Taking all trials and both disc entry and the transitioning between discs into account, the gamer group moved and tilted the exoscope camera less often than the control group, *p* = 0.01 (Figs. 3 and 5). Movement became more efficient in both groups, *p* = 0.01, and development did not differ significantly between the groups, *p* = 0.35.

Statistically significant differences between the two groups were not observed in the following variables: zoom, disc visibility and the location of the target discs on the viewing field (Figs. 3 and 5).

### Fine motor skills

The participants of both groups dropped the needle as often. Statistical significance was not indicated between groups, *p* = 0.66, but was indicated between trials, *p* = 0.024 (Fig. 3). Development did not differ significantly between groups, *p* = 0.31.

The participants of the control group took both instruments out of the optimal viewing field more often than the participants in the gamer group (Fig. 3). Statistical significance was indicated both between groups, *p* = 0.011, and trials, *p* = 0.0065. Development did not differ significantly between groups, *p* = 0.11.

## Discussion

Supporting our first hypothesis, long-term video gaming was associated with better initial adaptability to the exoscope. Gamers were more fluent with the camera motion; they were faster and needed fewer adjustments to reach optimal visualization of the target discs. This is probably explained by superior 3D perception among the gamers, which results in better exoscope utilization. However, it is also possible that gamers simply adapt faster to the exoscope foot pedal. The gamers drifted out of the surgical field with their forceps less often than the participants of the control group, which supports the hypothesis of the superior 3D perception in gamers. Both groups employed similar zoom.

Supporting our second hypothesis, all students improved during the training period. Novice users appear to adapt quickly to surgical exoscopes. Training, albeit as brief as in our study, is clearly beneficial and results in rapid improvement. There were no statistically significant differences in the learning curves of students from the two groups.

Supporting our third hypothesis, the gamers passed the needle through the discs in similar time than the students in the control group and both groups dropped the needle as often. It seems that video gaming results in minimal, if any, microsurgical manual benefits. A previous study also concluded that there was no correlation between video gaming or instrument playing and improved microsurgical performance in a group of 46 students [31]. In our study, the differences between the two groups are explained by better adaptability to the exoscope, not by differences in manual skills. Manual training with surgical instruments would be required to gain an advantage at the manual portion of the task.

The potential benefits of gaming experience have been investigated but little in the context of adapting to exoscopes. Calloni et al. [2] compared an exoscope to a traditional operative microscope in the identification of anatomic structures in a cranial approach model. They included 16 participants with at least 100 h of gaming experience. No statistically significant differences were found between gamers and non-gamers in the time taken to identify the structures under the exoscope. One reason explaining the result could be the limited amount of required gaming hours.

In our study, we categorized the participants based on their video gaming experience: those with over 1,000 h of gaming formed the gamer group, while individuals with less than 500 h comprised the control group. These thresholds were chosen to create a clear contrast between participants with extensive versus minimal gaming exposure. We avoided setting excessively high or low thresholds—e.g., 10,000 h—as such criterion would hinder recruitment and reduce the generalizability of the findings. In contrast, some previous studies have defined gamers as those with as little as 100 h of experience, a criterion that is easily met by most medical students in modern society. Our thresholds thus reflected a balance between scientific rigor, and practical feasibility. Participants whose gaming hours fell between 500 and 1,000 were excluded to preserve group integrity.

The significance of gaming experience has been investigated more extensively in laparoscopic, robotic and endoscopic surgery [11, 23]. Exoscope-assisted surgery combines some aspects of both robotic and laparoscopic surgery. When utilizing an exoscope, surgeons do not see their feet when maneuvering the camera with the foot pedal. Similarly, in robotic surgery, robotic arms are controlled manually out of the surgeon’s line of sight. Meanwhile, laparoscopic surgery requires surgeons to observe their actions on-screen, much like in exoscopic surgery. A positive correlation between the amount of video gaming experience and superior adaptation to robotic surgery and laparoscopy was reported in thirteen studies [1, 4, 6, 7, 12, 14, 16, 17, 21, 25, 26, 35]. Another twelve studies reported similar findings for endoscopy but the gamers did not acquire robotic techniques quicker [8, 9, 13, 15, 24, 32, 33, 36, 37, 41]. The complexity of a surgical task may dwarf the benefits of video gaming. In our study, the simple training model was able to capture significant differences between the two groups. However, future studies with more complex models should be conducted to understand whether these differences could persist in clinical scenarios. We do not suggest that video gaming experience makes a good surgeon. However, there are clear indications that virtual simulations, which could be accessed frequently and easily, would be helpful in surgical training. An easily accessible digital exoscope simulator, including a physical exoscope foot pedal and virtual 3 d brain surgery models could be highly beneficial to surgeons who are not familiar with the exoscope. We have begun working on such a simulator. In addition, short-term training courses on exoscope use might be beneficial for microsurgical novices prior to operating on a patient. Even six hours of training could be beneficial [39].

### Strengths

Our easily replicable standardized test model was one of the major strengths of this study, enabling measuring of both microsurgical dexterity and the ability to control the exoscope camera. The task of threading a needle through small plastic discs under an exoscope in tight angles is useful for assessing basic motor adaptation and exoscope manoeuvring ability. Multiple senior and resident neurosurgeons at the Helsinki University Hospital also found the model to be a helpful training tool, and a relatively challenging one, especially when time pressure was applied. Other training models to enhance 3D perception and hand–eye coordination are also available, ranging from accurate anatomical models to abstract models specifically designed to help surgeons get better at certain skills. In addition, many training models can be accessed by trainees with ease in a microsurgical laboratory or even without a microscope at home using methods as simple as a computer, smartphones, and virtual reality glasses [3, 5].

Second, good quality video recordings facilitated an in-depth analysis of the trials. Third, the novice participants had no prior experience with surgical microscopes or in surgery, and thus we were able to focus on the effects of video gaming.

### Limitations

We acknowledge some limitations. First, due to some issues with equipment, we failed to record the test runs of one student from the control group and another from the gamer group. However, we managed to record the time taken to complete all three trials of both students. Second, the study included twenty participants, limiting statistical analysis. Studies with larger sample size are required to confirm our results. Third, the long-term effects of video gaming on the adaptation to surgical exoscopes are not considered in this study. Fourth, the task runs were conducted in laboratory conditions among surgical novices, and the results cannot be directly applied to clinical practice. Last, the sample size of twenty introduces some variability and makes it difficult to confidently attribute the observed differences solely to gaming experience. However, the two groups were homogenous in multiple other aspects such as handicraft and 3d-modelling experience. Individual differences in visuospatial ability naturally will have impacted the results, however, video gaming experience itself increases depth perception [22]. Thus, individual differences in visuospatial ability are expected and necessary for the purposes of this study.

## Conclusions

Video gaming experience seems to facilitate adaptation to the surgical exoscope among novice users. Gamers appear to have superior 3D perception and better ability to adapt to the exoscope joystick. Short-term training resulted in significant improvements in exoscope handling among non-gamer participants as well, suggesting that even a brief introduction to the device in a laboratory setting is beneficial.

## Supplementary Information

Below is the link to the electronic supplementary material.

Supplementary file1 (MP4 21.7 MB)

## Data Availability

No datasets were generated or analysed during the current study.
